# Supplementary material for: Diffuse Optical Monitoring of Cerebral Hemodynamics and Oxygen Metabolism during and after Cardiopulmonary Bypass: Hematocrit Correction and Neurological Vulnerability
Source: Metabolites. 2023 Nov 16;13(11):1153. doi: 10.3390/metabo13111153 (PMC10672802; doi:10.3390/metabo13111153)
Supplement: Supplementary file 1 [file metabolites-13-01153-s001.zip › metabolites-2677899-supplementary.pdf]

Article

Diffuse Optical Monitoring of Cerebral Hemodynamics and Oxygen Metabolism During and After Cardiopulmonary Bypass: Hematocrit Correction and Neurological Vulnerability

Supplementary Materials

S1. Derivation of Hematocrit Corrected CBF

In prior work, Boas et. al. [S1] derived the following theoretical relation between CBF and the DCS BFI based on shear-induced diffuse motion of red blood cells [S2]:

$$CBF = \frac{3\, R\, \mu'_{s,avg}}{8\, \alpha_{shear}\, \mu'_{s,blood}} \times BFI\ . \tag{S1}$$

As defined in the main text, $\mu'_{s,avg}$ is the reduced scattering coefficient of tissue at the DCS light wavelength, R is the average radius of the blood vessels sampled by DCS, α_{shear} is the proportionality between the shear flow rate and the red blood cell diffusion coefficient (empirically observed to be in the range of 10^{-7} - 10^{-6} mm^2 [S2, S3]), and $\mu'_{s,blood}$ is the reduced scattering coefficient of blood. Note that $\mu'_{s,blood} = \mu_{s,blood}(1-g)$, where $\mu_{s,blood}$ is the scattering coefficient of blood and g is the scattering anisotropy coefficient of the scatterer within blood (i.e., the red blood cell). Note also that $\mu_{s,blood}$ is the product of the number density of red blood cells with the light scattering cross-section of a red blood cell, *i.e.*, $\mu_{s,blood} = \rho_{RBC} \times \sigma_{RBC}$. The number density of red blood cells in blood can be written in terms of the hematocrit (*Hct*): $\rho_{RBC} = Hct/vol_{RBC}$, where vol_{RBC} is the volume of a red blood cell. Thus, the reduced scattering coefficient for blood is

$$\mu'_{s,blood} = \frac{Hct}{vol_{RBC}} \times \sigma_{RBC}(1-g). \tag{S2}$$

Substituting Equation (S2) into Equation (S1), we obtain

$$CBF \approx \frac{3\ vol_{RBC} \times \mu'_{s,avg} \times R}{8\, \alpha_{shear} \times Hct \times \sigma_{RBC} \times (1-g)} \times BFI\ . \tag{S3}$$

S2. Impact of Accounting for PaO_2, Hct and $\mu'_{s,avg}$ in the calculation of CaO_2, OEF, CBF and $CMRO_2$

The hematocrit (*Hct*) correction of *in vivo* DCS BFI data, as described above, results in CBF scaling inversely proportionally with *Hct* and proportionally with $\mu'_{s,avg}$. In the following scenarios we examine the individual errors in CBF and $CMRO_2$ quantification associated with neglecting $\mu'_{s,avg}$ or *Hct* individually (Scenario 1 or Scenario 2, respectively), and together (Scenario 3). Additionally, we examine the incremental error associated with neglecting the contribution of PaO_2 (i.e., free dissolved arterial oxygen) in the calculation of CaO_2 and OEF (Scenario 4). Finally, we explore common scenarios where invasive arterial blood gas sampling to quantify *Hct* and PaO_2 is only available at baseline (Scenario 5), or not available at all (Scenario 6). The error associated with each scenario is calculated relative to the quantitative values achieved when PaO_2, *Hct*, and $\mu'_{s,avg}$ are accounted for in the calculation of mean CaO_2, OEF, CBF and $CMRO_2$ during baseline and during mild hypothermic CPB (Reference Scenario). Each scenario is explicitly defined as follows, and results summarized in **Table S1**:

- **Reference Scenario**. Repeated arterial blood gas sampling, CBF corrected for *Hct* and $\mu'_{s,avg}$, OEF and CaO_2 corrected for *Hct* and PaO_2. CBF correction for *Hct* and $\mu'_{s,avg}$ performed using Equation (8). CaO_2 correction for PaO_2 and *Hct* performed using Equation (4). CaO_2 correction for PaO_2 and *Hct* also impacts the calculation of OEF per Equation (5). OEF is further impacted by *Hct* correction via its dependency on CvO_2 which incorporates *Hct* per Equation (6).
- **Scenario 1**. CBF neglects $\mu'_{s,avg}$: CBF corrected for *Hct*, but not $\mu'_{s,avg}$.
- **Scenario 2**. CBF neglects *Hct*: CBF corrected for $\mu'_{s,avg}$, but not *Hct*.
- **Scenario 3**. Uncorrected CBF: CBF not corrected for $\mu'_{s,avg}$ and *Hct*.
- **Scenario 4**. Uncorrected CBF; neglect PaO_2: CBF not corrected for $\mu'_{s,avg}$ and *Hct*. OEF and CaO_2 only corrected for *Hct*, not PaO_2. This scenario may occur when there is only venous vascular access to a patient and changes in *Hct* may be corrected for based on repeated venous blood gas sampling.

- **Scenario 5**. Baseline blood gas only; uncorrected CBF: Arterial blood gas sampling at baseline only. Thus, only baseline OEF and CaO_2 corrected for *Hct* and PaO_2. PaO_2, *Hct*, and CaO_2 assumed to remain constant from baseline. CBF not corrected for $\mu'_{s,avg}$ and *Hct*.
- **Scenario 6**. Standard Analysis - No blood gas or hematocrit information; uncorrected CBF: No CaO_2 calculation. No OEF correction for *Hct* and PaO_2. *Hct*, and CaO_2 assumed to remain constant from baseline. CBF not corrected for $\mu'_{s,avg}$ and *Hct*. In this scenario, it is possible to estimate OEF as:

$$OEF = \frac{SaO_2 - StO_2}{\gamma SaO_2}, \tag{S4}$$

where γ is the fixed venous fraction also used in the estimation of SvO_2 in Equation (7). This scenario represents the conventional approach commonly used in clinical diffuse optical measurements of $CMRO_2$.

Table S1. Error in CMRO2 Parameters Associated with Correction for PaO_2, *Hct* and $\mu'_{s,avg}$

	**Baseline**		**Mild Hypothermic CPB**					
**Scenario**	CaO_2	OEF	CaO_2	OEF	$rCaO_2$	rOEF	rCBF	$rCMRO_2$
Reference (n=26)	10 [9, 12] (mL O_2/dL blood)	62 [56, 66] (%)	16 [15, 16] (mL O_2/dL blood)	55 [49, 58] (%)	165 [132, 180] (% Baseline)	88 [83, 98] (% Baseline)	64 [45, 90] (% Baseline)	91 [57, 135] (% Baseline)
**% Error**, Median [IQR]								
1. CBF neglects $\mu'_{s,avg}$	0.0 % [0.0, 0.0]	0.0 % [0.0, 0.0]	0.0 % [0.0, 0.0]	0.0 % [0.0, 0.0]	0.0 % [0.0, 0.0]	0.0 % [0.0, 0.0]	+1.4 % [-3.7, 5.1]	+1.4 % [-3.7, 5.1]
2. CBF neglects *Hct*	0.0 % [0.0, 0.0]	0.0 % [0.0, 0.0]	0.0 % [0.0, 0.0]	0.0 % [0.0, 0.0]	0.0 % [0.0, 0.0]	0.0 % [0.0, 0.0]	+58 % [26, 72]	+58 % [26, 72]
3. Uncorrected CBF	0.0 % [0.0, 0.0]	0.0 % [0.0, 0.0]	0.0 % [0.0, 0.0]	0.0 % [0.0, 0.0]	0.0 % [0.0, 0.0]	0.0 % [0.0, 0.0]	+57 % [31, 79]	+57 % [31, 79]
4. Uncorrected CBF + Neglect PaO_2	-2.8 % [-3.0, -2.3]	-1.7 % [-2.1, -1.3]	-4.9 % [-5.2, -4.6]	-4.2 % [-5.0, -3.6]	-2.2 % [-2.7, -1.6]	-2.4 % [-3.1, -1.8]	+57 % [31, 79]	+50 % [27, 72]
5. Baseline Blood Gas Only	0.0 % [0.0, 0.0]	0.0 % [0.0, 0.0]	-39 % [-44, -24]	-5.4 % [-6.7, -4.6]	-39 % [-44, -24]	-5.4 % [-6.7, -4.6]	+57 % [31, 79]	-9.6 % [-14, -4.9]
6. Standard Analysis – No PaO_2 or *Hct*	-	-1.7 % [-2.1, -1.3]	-	-8.0 % [-9.4, -6.8]	-39 % [-44, -24]	-6.4 % [-8.0, -4.9]	+57 % [31, 79]	-10 % [-15, -5.2]
Δ% **Error**, Median [IQR]								
Scenario 4 vs. 3 CaO_2, OEF neglect PaO_2	-2.8 % [-3.0, -2.3]	-1.7 % [-2.1, -1.3]	-4.9 % [-5.2, -4.6]	-4.2 % [-5.0, -3.6]	-2.2 % [-2.7, -1.6]	-2.4 % [-3.1, -1.8]	0.0 % [0.0, 0.0]	-6.7 % [-8.5, -4.9]
Scenario 6 vs. 4 CaO_2, OEF neglect *Hct*	-	0.0 % [0.0, 0.0]	-	-3.5 % [-4.6, -2.7]	-37 % [-44, -22]	-3.6 % [-4.7, -2.8]	0.0 % [0.0, 0.0]	-57 % [-76, -32]
Scenario 6 vs. 5 + Baseline *Hct*, PaO_2	-	-1.7 % [1.3, 2.1]	-	-2.3 % [2.1, 3.0]	-0.0 % [0.0, 0.0]	-0.8 % [0.4, 1.1]	0.0 % [0.0, 0.0]	-0.9 % [0.3, 1.2]

Error analysis of Scenario 2 versus Scenario 1 demonstrates the greater impact of neglecting *Hct* versus $\mu'_{s,avg}$ (+58 [26, 72] % vs. +1.4 [-3.7, 5.1] % error) on calculation of rCBF during mild hypothermia CPB; this error propagates into comparable errors in $rCMRO_2$. As a result, the error associated with neglecting both *Hct* and $\mu'_{s,avg}$ (i.e., uncorrected

CBF; Scenario 3) only modestly differs versus CBF correction neglecting *Hct* (Scenario 2) (+57 [31, 79] % vs. +58 [26, 72] % error).

Neglecting the contribution of PaO_2 (Scenario 4 versus Scenario 3), results in errors in CaO_2 and OEF at baseline and during mild hypothermic CPB. While the magnitude of errors at baseline (i.e., normative oxygen levels, PaO_2 = 91 [84, 96] mmHg) are reasonably small (<3%), error magnitude increases during hyperoxic CPB conditions (PaO_2 = 257 [237, 271] mmHg) for both CaO_2 (-4.9 [-5.2, -4.6] % vs. -2.8 [-3.0, -2.3] % error) and for OEF (-4.2 [-5.0, -3.6] % vs. -1.7 [-2.1, -1.3] % error). The combination of errors in OEF and CaO_2 propagates into a -6.7 [-8.5, -4.9] % effect on $rCMRO_2$ during CPB attributed to neglecting PaO_2.

The comparison of Scenario 6 versus Scenario 4 provides insights into the added effect of neglecting changes in *Hct* (as well as changes in PaO_2 already incorporated in Scenario 4) on CaO_2, OEF, and $rCMRO_2$ quantification when no blood gas information is available. The impact of neglecting *Hct* on OEF quantification during CPB (-8.0 [--9.4, -6.8] % vs. -4.2 [-5.0, -3.6] % error) is smaller than the impact on CaO_2 estimation. The assumption of constant CaO_2 (i.e., $rCaO_2$ = 1) during CPB results in a -39 [-44, -24] % error from the reference $rCaO_2$ value; this is a -37 [-44, -22] % change in error from Scenario 4 due to neglect of *Hct* in Scenario 6. Together this results in a combined median impact of -57 [-76, -32] % error on $rCMRO_2$ quantification. Note, this is comparable to the error in CBF associated with neglecting *Hct*. However, the negative errors in OEF and CaO2 offset the positive error from the use of uncorrected rCBF resulting in a reduction in the magnitude of $rCMRO_2$ error (+50 [27, 72] % error reduced to -10 [-15, -5.2] % error).

This compensatory effect on $CMRO_2$ error is apparent when examining *Hct* in the combined multiplicative expression for $CMRO_2$ (Equation (2), duplicated as Equation (S5) below). In this expression, CaO_2, which (when ignoring the additive contribution of PaO_2) scales proportionally with *Hct*, is multiplied by CBF, which scales inversely proportional to *Hct*. As a result, the *Hct* correction for each term cancels. This reduction follows from:

$$CMRO_2 = CaO_2 \times OEF \times CBF = CaO_2 \times \left(\frac{CaO_2 - CvO_2}{CaO_2}\right) \times CBF = (CaO_2 - CvO_2) \times CBF. \tag{S5}$$

Substituting in Equation (4) for CaO_2, Equation (6) for CvO_2, and Equation (8) for CBF, and substituting *A*, *B*, and *C* for assumed scalar constants defined as:

$$A = \left(1.39 \frac{mL\ O_2}{g\ Hgb}\right) \times \left(32.2\ \frac{g\ Hgb}{dL\ \ blood}\right), \tag{S6}$$

$$B = 0.003 \left(\frac{mL\ O_2}{dL\ blood \times mmHg}\right), \tag{S7}$$

$$C = \frac{3\ vol_{RBC}\ \times\ R}{8\ \alpha_{shear}\ \times\ \sigma_{RBC} \times (1-g)}, \tag{S8}$$

we arrive at:

$$CMRO_2 = \left(A \times (SaO_2 - SvO_2)\ +\ B \times \frac{PaO_2}{Hct}\right) \times\ \mu'_{s,avg} \times BFI \times C. \tag{S9}$$

Substituting in Equation (7) for SvO_2, results in an expression for $CMRO_2$ based on optical measurements StO_2, $\mu'_{s,avg}$, and BFI, and blood gas measurements SaO_2, PaO_2, and *Hct*:

$$CMRO_2 = \left(A \times \frac{SaO_2 - StO_2}{\gamma}\ +\ B \times \frac{PaO_2}{Hct}\right) \times\ \mu'_{s,avg} \times BFI \times C. \tag{S10}$$

Thus, even without knowledge of *Hct* or PaO_2 from blood gas, by using non-invasive pulse oximetry to estimate SaO_2 from SpO_2, it is possible to estimate $CMRO_2$ with marginal error; this error is attributed to the PaO_2/Hct term and its magnitude relative to the $(SaO_2 - SvO_2)$ term. Replacing the additive terms with the variables S and E, where:

$$S = A \times \frac{SaO_2 - StO_2}{\gamma}, \qquad E = B \times \frac{PaO_2}{Hct}, \tag{S11}$$

then the fractional error in $CMRO_2$ may be expressed as:

$$f_{error} = \frac{CMRO_{2,\text{uncorrected}} - CMRO_2}{CMRO_2} = \frac{S - (S+E)}{S+E} = -\frac{E}{S+E}. \tag{S12}$$

Because both S and E are necessarily positive, the fractional error will always be negative (i.e., $|f_{error}| = -f_{error}$). The error increases in magnitude with increasing PaO_2, decreasing Hct, decreasing SaO_2, or increasing SvO_2. In situations where PaO_2 and SaO_2 remain normoxic (PaO_2 between 80-100 mmHg), hematocrit is within our baseline interquartile range of 20-27%, and the difference $(SaO_2 - SvO_2)$ ranges between 54-65% (approximated from baseline OEF and SaO_2), then the fractional error of $CMRO_2$ ranges between 3-6%.

Examining the expression for relative $CMRO_2$ ($rCMRO_2$) compared to baseline values (denoted by a subscripted zero) the expression increases in complexity:

$$rCMRO_2 = \frac{CMRO_2}{CMRO_{2,0}} = \frac{\frac{A}{\gamma} \times (SaO_2 - StO_2) + B \times \frac{PaO_2}{Hct}}{\frac{A}{\gamma} \times (SaO_{2,0} - StO_{2,0}) + B \times \frac{PaO_{2,0}}{Hct_0}} \times \frac{\mu'_{s,avg}}{\mu'_{s,avg,0}} \times \frac{BFI}{BFI_0} = \frac{S+E}{S_0+E_0} \times \frac{\mu'_{s,avg}}{\mu'_{s,avg,0}} \times \frac{BFI}{BFI_0}. \quad (S13)$$

The fractional error of $rCMRO_2$ estimation which neglects the PaO_2/Hct terms (i.e., E and E_0, as defined above) follows as:

$$\frac{rCMRO_{2,\text{uncorrected}} - rCMRO_2}{rCMRO_2} = \frac{\frac{S}{S_0} - \frac{S+E}{S_0+E_0}}{\frac{S+E}{S_0+E_0}} = \frac{\frac{S}{S+E}}{\frac{S_0}{S_0+E_0}} - 1 = \frac{1+f_{error}}{1+f_{error,0}} - 1 = \frac{f_{error} - f_{error,0}}{1+f_{error,0}}. \quad (S14)$$

Given the small $CMRO_2$ errors anticipated under normoxic baseline conditions shown above, (i.e., $f_{error,0} \ll 1$), then $1 + f_{error,0} \approx 1$ and the expression simplifies to:

$$\frac{rCMRO_{2,\text{uncorrected}} - rCMRO_2}{rCMRO_2} \approx f_{error} - f_{error,0} = |f_{error,0}| - |f_{error}|. \quad (S15)$$

If there is no baseline $CMRO_2$ error (i.e., $f_{error,0} = 0$), then $rCMRO_2$ error is equivalent to $CMRO_2$ error (i.e., f_{error}). If baseline $CMRO_2$ error is greater than CPB $CMRO_2$ error (i.e., $|f_{error,0}| > |f_{error}|$), then $rCMRO_2$ error will be positive and result in an overestimation. However, if the reverse is true, i.e., error increases during CPB (as is the case in our analysis), then $rCMRO_2$ will be underestimated. Altogether, without Hct or PaO_2 information, we were able to estimate $rCMRO_2$ with -10 [-15, -5.2] % error during hyperoxic, mild hypothermic CPB.

Comparison of Scenario 5 versus Scenario 6 evaluates the utility of acquiring a single baseline blood gas and using knowledge of baseline PaO_2 and *Hct* to improve quantification error. This knowledge provides a small reduction in OEF error (-2.3 [2.1, 3.0] %), however the median impact on $rCMRO_2$ quantification is < 1%. Thus, the relative utility of a single blood gas timepoint is small.

Altogether, the analysis of the individual contributions of PaO_2, *Hct*, and $\mu'_{s,avg}$ demonstrate the predominant role of *Hct* correction of both CBF and CaO_2 to achieve best physiologic accuracy. Correction for free dissolved oxygen contributions associated with PaO_2 also resulted in significant, but relatively smaller, reductions in quantification error. Correction for $\mu'_{s,avg}$ was the least impactful correction. These results strongly support the use of repeated blood gas analysis in patient populations where large fluctuations in *Hct* or PaO_2 occur to improve diffuse optical quantification of CBF and $CMRO_2$.

S3. References

S1. Boas, D.A.; Sakadžic, S.; Selb, J.; Farzam, P.; Franceschini, M.A.; Carp, S.A. Establishing the diffuse correlation spectroscopy signal relationship with blood flow. *Neurophotonics* **2016**, *3*, 031412. https://doi.org/10.1117/1.nph.3.3.031412.

S2. Goldsmith, H.L.; Marlow, J.C. Flow behavior of erythrocytes. II. Particle motions in concentrated suspensions of ghost cells. *J. Colloid Interface Sci.* **1979**, *71*, 383–407. https://doi.org/10.1016/0021-9797(79)90248-0.

S3. Tang, J.; Erdener, S.E.; Li, B.; Fu, B.; Sakadzic, S.; Carp, S.A.; Lee, J.; Boas, D.A. Shear-induced diffusion of red blood cells measured with dynamic light scattering-optical coherence tomography. *J. Biophotonics* **2018**, *11*, e201700070. https://doi.org/10.1002/jbio.201700070.
